# Regular Exercise or Changing Diet Does Not Influence Aortic Valve Disease Progression in LDLR Deficient Mice

**DOI:** 10.1371/journal.pone.0037298

**Published:** 2012-05-14

**Authors:** Florian Schlotter, Yasuharu Matsumoto, Norman Mangner, Gerhard Schuler, Axel Linke, Volker Adams

**Affiliations:** 1 University of Leipzig – Heart Center Leipzig, Department of Internal Medicine/Cardiology, Leipzig, Germany; 2 Tohoku University Graduate School of Medicine, Department of Cardiovascular Medicine, Sendai, Japan; State University of Rio de Janeiro, Biomedical Center, Institute of Biology, Brazil

## Abstract

**Background:**

The development and progression of calcific aortic valve disease (CAVD) shares a number of similarities with atherosclerosis. Recently we could demonstrate that regular exercise training (ET) as primary prevention prevents aortic valve disease in LDL-receptor deficient (LDLR^−/−^) mice. We aimed to investigate the impact of exercise training on the progression of CAVD in LDLR^−/−^ mice in the setting of secondary prevention

**Methods and Results:**

Sixty-four LDLR^−/−^ mice were fed with high cholesterol diet to induce aortic valve sclerosis. Thereafter the animals were divided into 3 groups: group 1 continuing on high cholesterol diet, group 2 continuing with cholesterol diet plus 1 h ET per day, group 3 continuing with normal mouse chow. After another 16 weeks the animal were sacrificed. Histological analysis of the aortic valve thickness demonstrated no significant difference between the three groups (control 98.3±4.5 µm, ET 88.2±6.6 µm, change in diet 87.5±4.0). Immunohistochemical staining for endothelial cells revealed a disrupted endothelial cell layer to the same extend in all groups. Furthermore no difference between the groups was evident with respect to the expression of inflammatory, fibroblastic and osteoblastic markers.

**Conclusion:**

Based on the present study we have to conclude that once the development of a CAVD is initiated, exercise training or a change in diet does not have the potential to attenuate the progress of the CAVD.

## Introduction

Calcific aortic valve disease (CAVD) is a common medical condition in the elderly – up to 25% of adults over 65 have valvular sclerosis [1], and 2.8% of adults over 75 years old have some degree of CAVD [2], [3]. CAVD encompasses early sclerosis, characterized by leaflet thickening, to late stenosis in which leaflets stiffen, left ventricular outflow is obstructed, and cardiac function is compromised. As a consequence CAVD sclerosis is associated with a 50% increased risk of cardiovascular death and myocardial infarction [4], and the prognosis for patients with stenosis is very poor [5]. At present, no effective non-invasive therapy exists. Valve replacement, the second-most frequent indication for cardiac surgery [6], remains the only option to intervene, carrying a perioperative mortality of about 3% [7]. CAVD and atherosclerosis exhibit a common pathophysiology [8] including endothelial injury, macrophage infiltration and inflammation [9]. In addition, epidemiological studies also confirmed that aortic stenosis (AS) and atherosclerosis share several common risk factors, like older age, diabetes, smoking, hypertension and elevated levels of LDL [10]. For decades considered degenerative in nature, nowadays understanding of AV sclerosis and subsequent stenosis changed towards the perception of an active process that can possibly be modified [8], [11]. The early valvular changes exhibited by aortic valve sclerosis are considered to be modifiable by medical therapy in order to prolong the time until severe aortic stenosis develops and to delay the timing of surgery [11]. Increased physical activity proved effective in the prevention and treatment of atherosclerosis [12]. It was thus an intriguing question to ask whether exercise training (ET) would also be beneficial in stenotic aortic valve disease too. Recent evidence suggests that exercise training exerts its positive effects on calcific AV disease in the setting of primary prevention by preservation of the valvular endothelial cell layer, leading to a subsequent decrease in the recruitment of inflammatory cells, oxidative stress and proosteogenic pathways [13]. No data exist so far showing that this therapy is also effective in the setting of secondary prevention, which may more resemble the daily clinical challenge. Therefore, aim of the present study was to assess whether ET would be successful in altering the progression of a preexisting sclerotic valvular lesion.

## Methods

### Ethics Statement

All procedures were approved by the local council of animal research (Regierungspräsidium Leipzig, TVV 40/08).

### Animals

**Figure 1:**
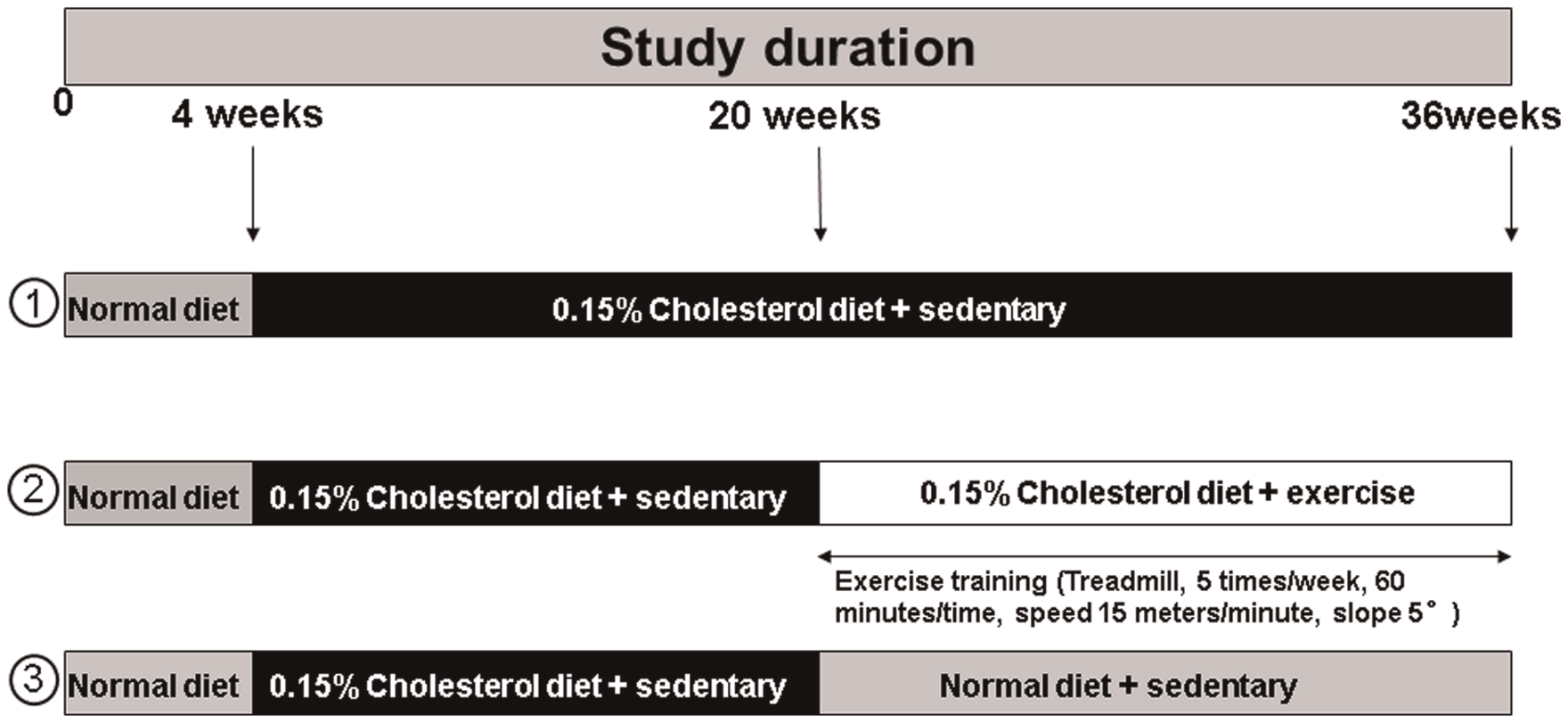
Experimental study design. Four-week old LDLR^−/−^ mice were fed cholesterol diet for 16 weeks. At week 20 all animals were randomized into one of the following groups: Group 1 -control group- (cholesterol diet plus sedentary activity); group 2 –exercise training group- (cholesterol diet plus exercise training); group 3 -change of diet group- (normal diet plus sedentary activity).

A total of 64 low-density lipoprotein (LDL)-receptor-deficient (LDLR^−/−^) mice on the C57BL/6J background at the age of 4 weeks were fed with cholesterol rich diet until 20 weeks. At 20 weeks, the mice were randomly divided into 3 groups: group 1 (control group), cholesterol diet plus sedentary lifestyle, group 2 (exercise group), cholesterol diet plus exercise training (ET) (1 h/day, 5 days/week), and group 3 (change of diet group), normal diet plus sedentary lifestyle. At an age of 36 weeks the animals were sacrificed by cervical dislocation and the tissue material was harvested for further analyses (Figure 1).

The cholesterol-rich diet (Western-type diet) contained 0.15% cholesterol and was obtained from Altromin GmbH (Lage, Germany). The animals were housed at the Animal Research Center of the University of Leipzig in a specific pathogen-free environment in rooms with a 7 am to 7 pm light/dark cycle.

### Training Protocol

Mice assigned to the ET group were taught to run on a motorized rodent treadmill with a shock-plate incentive. The slope of the treadmill was kept constant at 5°. Mice were trained at a speed of 15 m/min for 60 min/day with 2-minute rest intervals every 15 minutes, as described previously [13]. Except for the exercise period in the training group, all of the mice were confined to their cages throughout the study.

### Morphometric and Immunohistochemical Analysis

The hearts were fixed in 4% formalin for 24 hours. After fixation, the hearts were embedded into paraffin, and 4-µm serial sections were cut through the AVs. Sections showing all 3 cusps were stained with hematoxylin and eosin for general morphology. Overall thickness of the leaflets was averaged over 5 equally distributed length measurements per leaflet throughout the valve and quantified with an imaging software (Analysis 3.0, Olympus Soft Imaging Solutions GmbH, Münster, Germany) as previously described [13]. Immunohistochemistry for endothelial nitric oxide synthase (eNOS) (rabbit polyclonal anti-mouse NOS-3 antibody, Santa Cruz Biotechnology, Inc. Santa Cruz, USA), macrophages (rat monoclonal antibody against mouse Mac3, BD Biosciences, San Jose, USA), myofibroblast (α-smooth muscle actin (αSMA), 1A4, Sigma-Aldrich, Deisenhofen, Germany) and osteopontin (rabbit polyclonal anti-osteopontin, Acris Antibodies, Herford, Germany) was performed as previously described [13]. Endothelial cell layer integrity and intensity of immunohistochemical staining was quantified as previously described [13]. For histochemical detection of angiotensin II receptor subtype 1 expression a specific antibody (Santa Cruz Biotechnology, Inc. Santa Cruz, USA) was used a dilution of 1∶200.

Mineralization was visualized by von Kossa staining as recently described [13].

### Functional Assessment of the Aortic Valve by Echocardiography

To evaluate the AV function, echocardiographic assessment of AV flow velocity was performed at the day of sacrifice. Transthoracic echocardiography was performed with the Sonos 5500 echocardiogram (Agilent Technologies, Santa Clara, CA USA) equipped with a 12-Mhz phased-array transducer. The anterior thorax was shaved to optimize the acoustic interface. Warmed gel was applied, and the animal was gently cradled in the left lateral recumbent position. AV flow velocity was evaluated by continuous waves recorded through a near apical approach, and 5 beats were averaged. In 10 randomly selected mice, the inter-observer and intra-observer variability for the measurement of AV flow velocity were 3.7±1.1% and 1.9±0.3% respectively.

### Blood Biochemical Analysis

Serum was collected from sacrificed animals and stored at −80°C for further analyses. Serum levels of total cholesterol were determined enzymatically using a calorimetric assay kit (EnzyChrom Cholesterol AssayKit, ECCH-100, Bioassay Systems, Hayward, USA) as recommended by the manufacturer. The samples were assayed in duplicate.

### RNA-Isolation and Quantitative Assessment of mRNA Expression

AV cusps were scratched off from frozen sections on glass slides under microscopic control and gene expression of BMP-2 and α-smooth muscle actin was assessed as described earlier [13].

### Superoxide Detection

To examine the involvement of oxidative stress in the AVs, production of free radicals was evaluated using in situ dihydroethidium (DHE) fluorescence as previously described [13]. Briefly, frozen sections of aortic valves (30 µm) from the different groups were incubated at the same time with DHE (10 µmol/L) in PBS for 30 minutes at 37°C in a humidified chamber protected from light. DHE is oxidized on reaction with O2− to ethidium bromide, which binds to DNA in the nucleus and fluoresces red. Tissue sections were then visualized with an Axioplan-2 fluorescence microscope (Zeiss, Oberkochen, Germany) equipped with an Axio Cam MRC5 (Zeiss, Oberkochen, Germany). Frozen sections from all groups were processed in parallel, and images were acquired with identical acquisition parameters.

### Sample-Size Calculation

The primary end point of the study was AV thickness at 36 weeks of age. To calculate the sample size, it was hypothesized on the basis of a recent study [13] that AV thickness (mean±SD) of the control, ET and change of diet groups was 80±15 µm, 55±15 µm and 55±15 µm, respectively. Choosing a power of 90% and 5% type I error, we calculated that a sample size of at least 8 animals in each subgroup would be required to detect significant differences in AV thickness by ANOVA.

### Statistical Analysis

SPSS version 16.0 (SPSS Inc, Chicago, Ill) was used for all of the analyses. Data are expressed as mean±SEM. Comparisons among groups were performed with ANOVA. When data were not normally distributed or the variance was not equal, the Kruskal-Wallis nonparametric test was applied. A value of p<0.05 was considered statistically significant. All of the measurements were made by investigators blinded to the treatment group.

## Results

### Influence of Exercise Training on AV Diameter in Secondary Prevention

**Figure 2:**
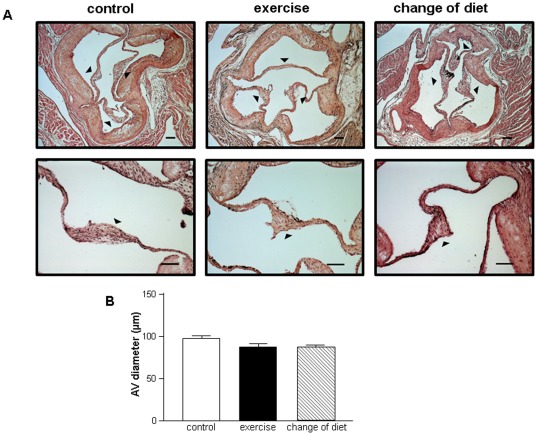
Tricuspid aortic valves (AVs) in LDLR^−/−^ mice stained with hematoxylin-eosin (A). Arrowheads indicate sites of AV leaflets. Bar = 100 µm. Quantitative analysis of overall AV thickness in 10 animals from each group (B).

Quantitative evaluation of AV diameter revealed that exercise training over a period of 16 weeks had no significant effect on the progression of AV thickening in secondary prevention in comparison with the control group (control group 98.3±4.5 µm, exercise group 88.2±6.6 µm; p = n.s.). Also in the “change in diet group" receiving normal rodent chow from the age of 20 weeks, no significant change in AV diameter progression was evident when compared to the other 2 groups (change of diet group 87.5±4.0 µm) (Figure 2).

### Effects of Exercise Training on Endothelial Cell Layer Integrity

**Figure 3:**
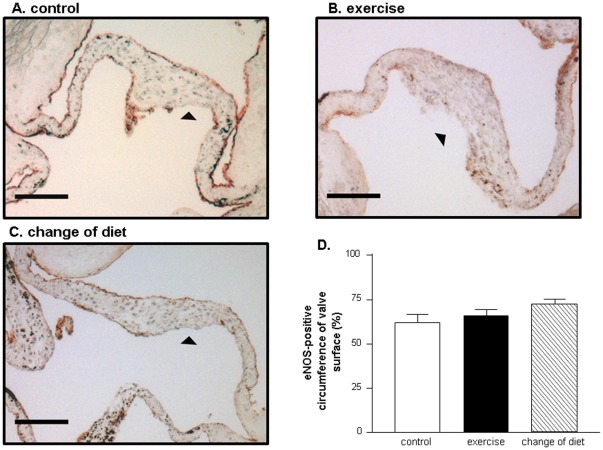
Representative immunostaining for endothelial nitric oxide synthase (eNOS) in all three animal groups. (A) Cholesterol diet plus sedentary activity (control group); (B) cholesterol diet plus exercise training; (C) normal diet plus sedentary activity. Quantitative analysis of eNOS-positive circumference in 9 animals per group (D). Arrowheads indicate disrupted area of eNOS expression. Bar = 100 µm.

In the control animals 62.6±5.4% of the AV surface was covered with endothelium as evident by eNOS-positive stained cells. An exercise training program showed no significant impact on the degree of endothelial cell coverage (exercise group 66.2±4.3%). The cholesterol-diet induced disruption of the endothelial layer could also not be preserved significantly by a change of diet (change in diet group 72.7±3.4%) (Figure 3).

### Markers of Inflammation and oxidative stress

**Figure 4:**
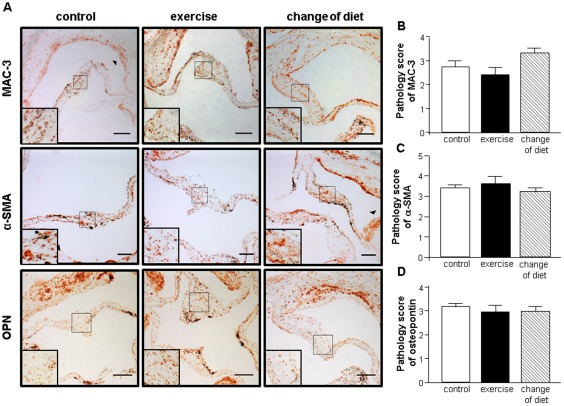
Representative immunostainings for macrophages (Mac3), vascular smooth muscle cells and valvular myofibroblast-like cells (α-smooth muscle actin [α-SMA]), and a phenotypic marker of osteoblast differentiation (osteopontin [OPN]) (A). Semi-quantitative analysis of infiltration of macrophages (B), valvular myofibroblast-like cells (C), and osteoblast-type cells (D) in 9 animals from each group. Arrowheads demonstrate areas of positive immunostaining at the vascular smooth muscle cell layer (α-SMA) and the atherosclerotic areas of the aortic sinus (Mac3). Bar = 100 µm.

Immunostaining for Mac-3, a marker for the presence of macrophages, was performed. Semi-quantitative analysis revealed a high level of macrophage infiltration of the AV leaflets that was almost identical among the three groups (Figure 4). Oxidative stress levels were assessed by in-situ Dihydroethidium (DHE) fluorescence. No significant difference in the degree of superoxide expression was observed between the groups (data not shown). Therefore, exercise training or a change in diet had no significant impact on the inflammatory process or the load with reactive oxygen species inside the AVs.

### Influence on the Level of Pro-calcific Signaling

**Figure 5:**
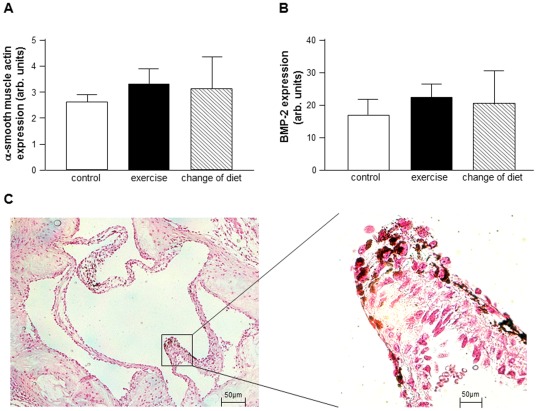
Quantitative analysis of messenger RNA expression for: α–smooth muscle actin, bone morphogenetic protein-2 (BMP-2, a mediator of calcification) (B) from aortic valve cusps of 8 animals from each group. arb. Units = arbitrary units. An example of a von Kossa staining is shown (C) (mineralization is shown as black dots).

α-smooth muscle actin-positive myofibroblasts were abundant inside the AVs of all three study groups. All AV leaflets showed a high level of α-smooth muscle actin-positive cells demonstrating fibrotic transformation. Semi-quantitative analysis revealed no significant differences between the 3 groups (Figure 4). Analysis of the m-RNA expression of α-smooth muscle actin confirmed the results of the immunohistochemical analyses with no significant differences observable between all study groups (control: 2.6±0.3 arb. units, exercise: 3.3±0.6 arb. units, change of diet: 3.1±1.3 arb. units; p = n.s.) (Figure 5A).

Moreover, osteopontin-positive cells could be detected in all three study groups, demonstrating a high degree of osteoblast differentiation (Figure 4). Semi-quantitative analysis exhibited no significant differences between the 3 study groups. To confirm this result, quantification of bone morphogenetic protein-2 by RT-PCR was performed. There was also no significant difference among the 3 groups (control: 17.3±4.8 arb. units, exercise: 22.6±4.4 arb. units, change of diet: 20.5±10.7 arb. units; p = n.s.) (Figure 5B). In addition, a weak staining for calcium deposits using the von Kossa stain was evident in the thickened part of the aortic valve leaflet (Figure 5C). Unfortunately a quantification of the weak signal in all three groups was not possible, while it was consistently positive over all the sections that were stained.

### Aortic Flow Velocity

Echocardiographic evaluations of maximum AV flow velocity at the age of 20 and 36 weeks, revealed a significant increase in all 3 groups over time (control 20 weeks: 1.26±0.06 m/s, control 36 weeks: 1.43±0.06 m/s; exercise 20 weeks: 1.18±0.03 m/s, exercise 36 weeks: 1.31±0.03 m/s; change of diet 20 weeks: 1.11±0.02 m/s, change of diet 36 weeks: 1.20±0.03 m/s). However, comparing the increase in maximum AV flow velocity over time (20 weeks vs. 36 weeks) showed no significant difference between the 3 groups. A regular flow pattern was observed as recently described [13].

### Exercise Training Reduces AT1 Expression at AVs

**Figure 6:**
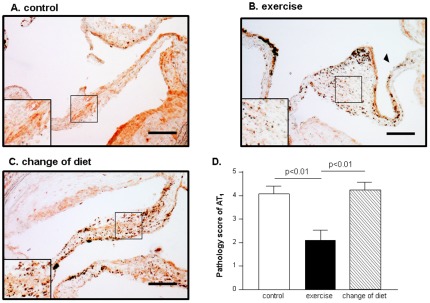
Representative immunostaining for angiotensin II receptor type 1 (AT1) in the control group, the cholesterol diet plus exercise training group and the normal diet plus sedentary activity (A–C). Semi-quantitative analysis of AT1-positive cells in 8 animals from each group (D). Arrowheads indicate positive staining of the endothelium serving as internal positive control. Bar = 100 µm.

The angiotensin II receptor type 1 (AT1), described as a further element of the pathologic changes inside the AV leaflets, was markedly down-regulated in its expression in those animals receiving exercise training. Semi-quantitative analysis showed a significantly lower expression of AT1 in the exercise group, while AVs of the control group and change of diet group exhibited high degrees of expression of AT1 (Figure 6).

### Effects on Body Weight and Lipid Metabolism

Analyzing the absolute change in body weight over the intervention period of 16 weeks, revealed a significantly higher increase in body weight in the control group, when compared to the exercise and change of diet group (control: 7.37±0.94 g; exercise: 3.70±0.46 g; change in diet: 3.69±0.75 g; p<0.01).

ET for a period of 16 weeks in secondary prevention did not significantly affect serum cholesterol levels in LDLR^−/−^ mice fed a cholesterol diet (control group 954.1±68.5 mg/dL, exercise group 1024.9±88.8 mg/dL). In contrast the animals that underwent a change in diet had markedly lower serum cholesterol levels (change in diet group 315.7±29.0 mg/dL).

## Discussion

This study is the first to examine effects of exercise training on the course of a preexisting sclerotic aortic valve lesion in a mouse model (secondary prevention).

The following novel findings emerge from this study: On the basis of a preexisting aortic valve sclerosis, ET or changing diet has no influence on thickening of AV leaflets or the increase in aortic flow velocity over time; ET or changing diet has no effect on endothelial cell layer integrity; ET or changing diet has no influence on molecular markers of inflammation and calcification.

The results of this study imply that AV sclerosis is a progressive process and exercise training has no significant effects on disease progression in secondary prevention.

### Impact of Exercise Training on Morphologic and Functional Features of the AV Leaflets

In a recent editorial Otto considers the direct evaluation of tissue changes inside the AV leaflets the ideal end point for measuring the effects of therapy [14]. Therefore, in our study the AV leaflet thickness served as the primary end point. In contrast to a primary prevention approach, as recently described by our group [13], secondary prevention by exercise training was ineffective in slowing down the progression of AV leaflet thickening. Comparing the results of the AV thickness collected in the present study, aiming at secondary prevention by ET, to those measured by Matsumoto et al. targeting primary prevention with the same ET program at an earlier intervention period [13], we clearly see a further increase of AV leaflet diameter over time (∼50 µm after 16 weeks vs. ∼90 µm after 32 weeks high cholesterol feeding). Even removing the high concentration of the atherogenic substrate cholesterol by changing the diet after the induction of AV alterations could not halt or slow down the progression of the morphological changes. These results are in agreement with the recently published results from randomized clinical studies using statins [15], [16], [17]. In one of these studies, the treatment of asymptomatic patients with mild to moderate aortic stenosis with rosuvastatin 40 mg did not reduce the progression of AS despite a reduction in cholesterol levels [15]. The ineffectiveness of both interventions, regular exercise training or changing diet, to alter the progressive nature of AV stenosis is supported by our functional measurements. Also by echocardiographic determination of maximum AV flow velocity, no differences between the intervention groups and the control group was evident. One possible explanation of the negative result may be the inappropriate training duration. Nevertheless, in primary prevention the same training protocol was very effective in preventing the initiation of aortic valve sclerosis [13]. In addition, we could clearly demonstrate an effect of the chosen training protocol on surrogate markers like body weight and the valvular expression of angiotensin II receptor type 1, which is known to be influenced by exercise training [18].

### Impact of Interventions on the Endothelium

The deterioration of the endothelial cell layer appears to be the initializing lesion in calcified AV disease and facilitates the activation of the pathologic cascade comprising macrophage infiltration, oxidized lipid deposition, myofibroblast differentiation and subsequent calcification [19]. The mechanical properties of AVs are actively regulated by endothelium-dependent mediators, such as NO and endothelin-1 through modulation of the contractile potential of valve interstitial cells [20]. The matrix stiffness of AVs determines the extend of calcific nodule formation- as the basis of calcification [21]. In case of failure to adapt the valve rigidity to different hemodynamic conditions, the resulting turbulent flow could enforce further AV endothelial cell layer alteration. Inability to restore endothelial integrity would thereby lead to a vicious cycle consisting of continued and accelerated inflammation, fibrosis and subsequent calcification. We have recently shown that preservation of the endothelial integrity by exercise training facilitates a conservation of the AV thickness [13]. Exercise training in secondary prevention was ineffective in restoring the endothelial cell layer in the present study and proved to be unable to influence downward pathobiologic elements of the disease process.

Independent from phenotypic variations between vascular and valvular endothelial cells [22], the differences in microenvironment between the one subtype that is exposed to laminar flow conditions in the vasculature and the other that is subjected to turbulent flow conditions at the aortic side of the valve could provide an explanation for the differences in ET efficacy in protecting the endothelial layer once pathologic changes have occurred.

### Impact on Secondary Outcome Measures

The level of macrophage infiltration, myofibroblast differentiation, oxidative stress and osteoblast phenotypes remained high, despite of exercise training or change in diet. These results obtained by specific immunohistochemical staining were further supported by quantifying the mRNA expression of BMP-2 (pro-calcification) and α-SMA (myofibroblast). Taken together all of these elements of the pathologic cascade leading to overt thickening and calcification of the AVs are increased in all subgroups of the present study and neither exercise training nor changing the diet had any influence on these molecular markers.

The serum cholesterol concentration significantly decreased after changing the diet of the animals after 16 weeks of Western type diet. Unfortunately, this did not result in slowing down disease progression. This is in contrast to a recently published study by Miller and colleagues [23]. In their study they reported that a significant reduction of the plasma cholesterol by knocking down the microsomal triglyceride transfer protein halts the progression of aortic valve stenosis. One possible explanation for this difference might be the approach for reducing the cholesterol level – genetic versus reduction of cholesterol intake.

It is well documented in the current literature, that components of the renin-angiotensin-aldosteron system (RAAS) are expressed at a higher level in stenotic aortic valves when compared to normal valves [11], [24], [25]. In the present study we also could document a robust expression of the AT1 receptor in the aortic valve leaflet after cholesterol rich diet, which was significantly down-regulated after 16 weeks of ET. Unfortunately, this exercise-induced reduction in AT1 expression had no impact on disease progression. Therefore, one may speculate that modulating components of the RAAS does not translate into a reduced progression of the disease. This notion is supported by a study of O'Brien and colleagues treating patients with mild to moderate AS with ramipril [24]. In that small study no impact of ramipril treatment on aortic jet velocity could be documented.

### Possible Explanations for the Failure of Secondary Prevention to Alter Disease Progression

As documented in the present study, the progression of AS can not be altered either by ET or changing the diet, once initiated. This finding is in line with the failure to provide conclusive evidence on the restoration or attenuation of disease progress by multiple pharmacological interventions like statins [15] or ACE inhibitors [26].

Even though CAVD is considered an active, possibly modifiable process, it remains an intriguing question to ask whether the tissue changes are subject to a fixation at the molecular level and if there is a putative point of no return as recently discussed by Sider and colleagues [27]. This could in part be explained as recently discussed [28] by epigenetic modification like methylation of key genes of the molecular pathways of the pathologic cascade, rendering any future intervention unsuitable to change the fate of the disease process unless these modifications can be removed from the promoter/enhancer/silencer regions of the genome of the valvular cells. Another possible explanation would be that, as discussed above, unfavorable flow conditions on the AV anticipating the known beneficial effect of ET on restoration of the endothelial cell layer. Nevertheless, both hypotheses need further molecular investigations.

### Study Limitations

Several limitations of the present study should be mentioned.

First, a regular control group without cholesterol feeding for 32 weeks is missing in the present study. So it may be difficult to make some conclusions about the severity of the AV sclerosis. Nevertheless, based on our previous study [13], where animals were fed with high cholesterol diet for 16 weeks, we see a clear progression of AV sclerosis in the cholesterol fed group and no impact on AV sclerosis by exercise training or changing diet, after the initiation (16weeks) of sclerotic modifications. Second, a group starting exercise training after 16 weeks cholesterol feeding and changing back to normal mouse chow was not investigated in the study. Based on the observation, that not even a trend towards slowing disease progression by the single interventions was observed, we speculated that a cumulative effect would also be negative. Nevertheless, proof of this speculation needs further investigation.

Third, an evaluation of myocardial function either by echocardiography or invasive measurement after induction of AV sclerosis and after finishing the intervention period is missing. Therefore, we can not make any conclusion on changes in myocardial performance after our interventions.

### Conclusion

Based on the present study in a selected animal model we have to conclude that once the initiation of the pathologic lesion is completed, exercise training or a change in diet does not have the potential to attenuate the progress of the AV disease process. Therefore one may speculate that for clinical approaches alternative therapeutic concepts to slow down the progression of aortic valve disease should be explored.

## References

[1] Beckmann E, Grau JB, Sainger R, Poggio P, Ferrari G (2010). Insights into the use of biomarkers in calcific aortic valve disease.. J Heart Valve Dis.

[2] Nkomo VT, Gardin JM, Skelton TN, Gottdiener JS, Scott CG (2006). Enriquez-Sarano M. Burden of valvular heart diseases: a population-based study.. Lancet.

[3] Writing Committee, Bonow RO, Carabello BA, Chatterjee K, de Leon AC, et al. (2008). 2008 Focused update incorporated into the ACC/AHA 2006 guidelines for the management of patients with valvular heart disease.. Circulation.

[4] Otto CM, Lind BK, Kitzman DW, Gersh BJ, Siscovick DS (1999). Association of aortic-valve sclerosis with cardiovascular mortality and morbidity in the elderly.. N Engl J Med.

[5] Ben-Dor I, Pichard AD, Gonzalez MA, Weissman G, Li Y (2010). Correlates and causes of death in patients with severe symptomatic aortic stenosis who are not eligible to participate in a clinical trial of transcatheter aortic valve implantation.. Circulation.

[6] Roberts WC, Ko JM (2005). Frequency by decades of unicuspid, bicuspid, and tricuspid aortic valves in adults having isolated aortic valve replacement for aortic stenosis, With or without associated aortic regurgitation.. Circulation.

[7] Gummert JF, Funkat A, Beckmann A, Schiller W, Hekmat K (2010). Cardiac surgery in Germany during 2009. A report on behalf of the German Society for Thoracic and Cardiovascular Surgery.. Thorac Cardiovasc Surg.

[8] Freeman RV, Otto CM (2005). Spectrum of calcific aortic valve disease.. Circulation.

[9] Newby DE, Cowell SJ, Boon NA (2006). Emerging medical treatments for aortic stenosis: statins, angiotensin converting enzyme inhibitors, or both?. Heart.

[10] Stewart BF, Siscovick D, Lind BK, Gardin JM, Gottdiener JS (1997). Clinical factors associated with calcific aortic valve disease.. J Am Coll Cardiol.

[11] Rajamannan NM (2009). Calcific aortic stenosis.. Arterioscler Thromb Vasc Biol.

[12] Thompson PD, Buchner D, Pina IL, Balady GJ, Williams MA (2003). Exercise and physical activity in the prevention and treatment of atherosclerotic cardiovascular disease: a statement from the Council on Clinical Cardiology (Subcommittee on Exercise, Rehabilitation, and Prevention) and the Council on Nutrition, Physical Activity, and Metabolism (Subcommittee on Physical Activity).. Circulation.

[13] Matsumoto Y, Adams V, Jacob S, Mangner N, Schuler G (2010). Regular exercise training prevents aortic valve disease in LDLR deficient mice.. Circulation.

[14] Otto C (2008). Calcified aortic stenosis – time to look more closely at the valve.. N Engl J Med.

[15] Chan KL, Teo K, Dumesnil JG, Ni A, Tam J, for the ASTRONOMER Investigators (2010). Effect of lipid lowering with rosuvastatin on progression of aortic stenosis.. Circulation.

[16] Rossebo AB, Pedersen TR, Boman K, Brudi P, Chambers JB (2008). Intensive llipid lowering with Simvastatin and Ezetimibe in aortic stenosis.. N Engl J Med.

[17] Cowell SJ, Newby DE, Prescott RJ, Bloomfield P, Reid J (2005). A Randomized Trial of Intensive Lipid-Lowering Therapy in Calcific Aortic Stenosis.. N Engl J Med.

[18] Adams V, Linke A, Kränkel N, Erbs S, Gielen S (2005). Impact of regular physical activity on the NAD(P)H oxidase and angiotensin receptor system in patients with coronary artery disease.. Circulation.

[19] Aikawa E, Nahrendorf M, Sosnovik D, Lok VM, Jaffer FA (2007). Multimodality molecular imaging identifies proteolytic and osteogenic activities in early aortic valve disease.. Circulation.

[20] El-Hamamsy I, Balachandran K, Yacoub MH, Stevens LM, Sarathchandra P (2009). Endothelium-dependent regulation of the mechanical properties of aortic valve cusps.. J Am Coll Cardiol.

[21] Yip CYY, Chen JH, Zhao R, Simmons CA (2009). Calcification by valve interstitial cells is regulated by the stiffness of the extracellular matrix.. Arterioscler Thromb Vasc Biol.

[22] Butcher JT, Tressel S, Johnson T, Turner D, Sorescu G (2006). Transcriptional profiles of valvular and vascular endothelial cells reveal phenotypic differences.. Arterioscler Thromb Vasc Biol.

[23] Miller JD, Weiss RM, Serrano KM, Brooks RM, Berry CJ (2009). Lowering plasma cholesterol levels halts progression of aortic valve disease in mice.. Circulation.

[24] O'Brien KD, Shavelle DM, Caulfield MT, McDonald TO, Olin-Lewis K (2002). Association of angiotensin-converting enzyme with low-density lipoprotein in aortic valvular lesions and in human plasma.. Circulation.

[25] Helske S, Lindstedt KA, Laine M, Mäyränpää M, Werkkala K (2004). Induction of local angiotensin II-producing systems in stenotic aortic valves.. J Am Coll Cardiol.

[26] Salas MJ, Santana O, Escolar E, Lamas GA (2011). Medical therapy for calcific aortic stenosis..

[27] Sider KL, Blaser MC, Simmons CA (2011). Animal models of calcific aortic valve disease..

[28] Miller JD, Weiss RM, Heistad DD (2011). Calcific aortic valve stenosis: Methods, models, and mechanisms.. Circ Res.

